# From STEMI to saddle pulmonary embolism: Management challenges and the role of VA-ECMO

**DOI:** 10.21542/gcsp.2026.4

**Published:** 2026-02-28

**Authors:** Enad Haddad, Irfan Ahsan, Sudeep Nugooru, Gregary D. Marhefka

**Affiliations:** 1 Department of Internal Medicine, Jefferson Abington Hospital, Abington, Pennsylvania; 2 Division of Cardiology, Department of Medicine, Thomas Jefferson University Hospital, Philadelphia, Pennsylvania

## Abstract

Acute pulmonary embolism and acute myocardial infarction are critical conditions with overlapping presentations, and their coexistence can be catastrophic. We report a 45-year-old man with prior mid LAD stent who presented with chest pain and ventricular fibrillation arrest, diagnosed with inferior/posterior STEMI. Emergent PCI to the mid RCA restored stability. The patient developed persistent hypoxia over the next two days requiring intubation and mechanical ventilation. Worsening hypoxia and shock prompted urgent transesophageal echocardiography to evaluate for mechanical complications, instead revealing a mobile echo density spanning the right and left main pulmonary arteries, consistent with acute saddle pulmonary embolism, and right ventricular dilation and hypokinesis. Due to rapidly progressive hypoxia and shock, he was placed on VA-ECMO and started on anticoagulation, improving without the need for urgent interventional or surgical embolectomy. This case underscores the importance of considering acute pulmonary embolism in post-myocardial infarction patients with unexplained hypoxia or shock, where overlapping features may delay diagnosis. Early VA-ECMO initiation can provide a bridge to recovery in such critically ill patients.

## Introduction

Pulmonary Embolism (PE) is a common life-threatening disease that requires prompt diagnosis and treatment. Yet, it is not infrequently missed or misdiagnosed^1^. Occasionally, its clinical presentation may mimic that of acute myocardial infarction (MI) or its complications, or even more rarely, overlap with it. In those cases, early recognition and intervention are crucial to improve outcomes and help prevent complications. We present a case of a 45-year-old male who was initially managed for acute MI secondary to mid RCA occlusion who later developed an acute saddle PE complicated by acute hypoxia and obstructive shock requiring VA-ECMO.

## Case presentation

**Figure 1.**
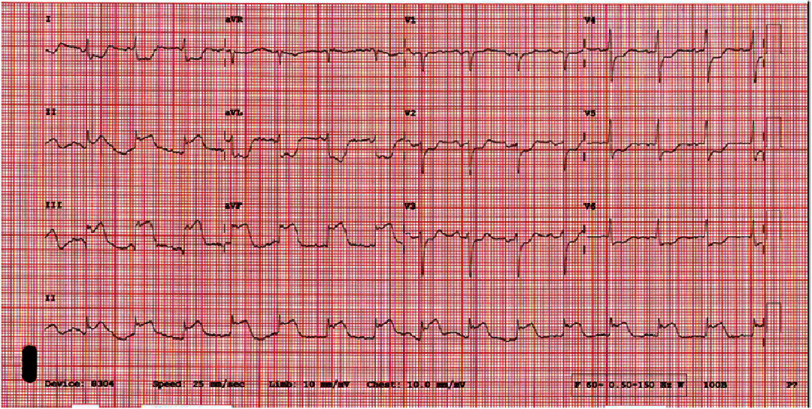
ECG showing and inferior/posterior STEMI along with reciprocal ST segment depression in the lateral leads.

The patient was a 45 year old male with a past medical history of coronary artery disease with a drug eluting stent to the mid LAD 4 years earlier for anterior STEMI, ischemic cardiomyopathy with a recovered ejection fraction, morbid obesity (body-mass index 41.8 kg/m^2^), hypertension, hyperlipidemia, and prediabetes, who called the ambulance after experiencing left sided chest pain for 2 h radiating to the left arm with associated diaphoresis (see timeline Figure 5). He was given aspirin and nitroglycerin by the emergency medical personnel. En route to the emergency department, the patient developed cardiac arrest secondary to ventricular fibrillation and underwent cardiopulmonary resuscitation and advanced cardiac life support including three external defibrillation shocks, with return of spontaneous circulation. On arrival, his electrocardiogram (ECG) showed inferior/posterior STEMI, with reciprocal ST segment depression in the lateral leads (Figure 1). Highly sensitive Troponin T level was 59 ng/L (normal range 0–19 ng/L). D-dimer level was not measured.

He was taken immediately to the cardiac catheterization laboratory and underwent a successful percutaneous coronary intervention of a 100% occlusion of the mid RCA with a drug eluting stent. His prior mid LAD stent was patent. He was loaded with prasugrel and intravenous tirofiban.

Post-procedure, the patient had persistent 3/10 chest pain and tenderness which was attributed to cardiopulmonary resuscitation. Echocardiogram the next day showed low-normal left ventricular function, with inferior and inferoseptal akinesis, ejection fraction 55% using echo contrast-enhanced imaging, no mitral regurgitation; the right ventricle was not well seen.

Two days later, the patient developed hypoxia, felt to be due to right middle and lower lobe collapse based on chest X-ray, for which he was placed on noninvasive mechanical ventilation and chest physiotherapy initiated.

The following day, hypoxia progressed and he required intubation and placement on the ventilator. He remained hypoxic, saturating between 80 and 82%. Bronchoscopy was performed and showed no evidence of obstructive airways or mucous plugging to explain the severe hypoxia. Repeat transthoracic echocardiogram did not reveal any changes or significant complications of MI and agitated saline injection was negative for intracardiac shunting, though again the right ventricle was not well visualized.

**Figure 2.**
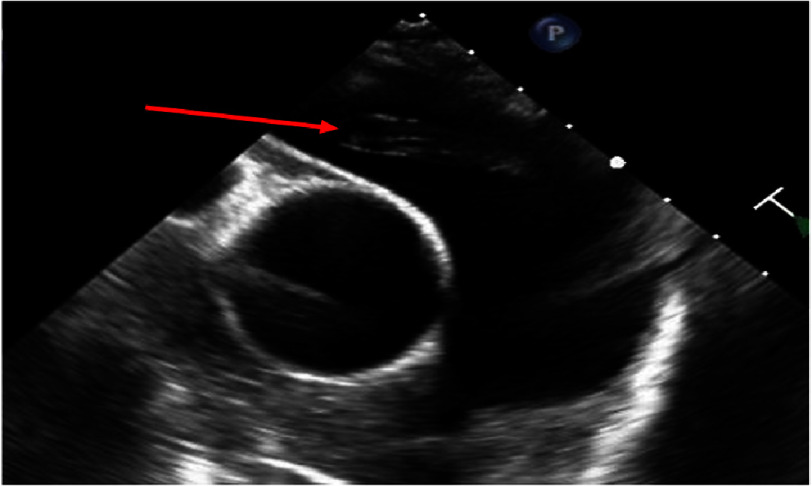
Transesophageal echocardiogram showing a long, mobile, serpiginous echodensity (arrow) from the pulmonary artery bifurcation to the right main pulmonary artery consistent with acute saddle PE.

Shortly after intubation, he developed hypotension and progressive shock despite being on intravenous norepinephrine and vasopressin infusions. ECG was unchanged compared to immediate post drug-eluting stent placement a few days earlier. D-dimer level was elevated at 2718 ng/mL (normal range 0–243 ng/mL); repeat highly sensitive troponin T was not performed. Despite the unrevealing transthoracic echocardiogram for acute mechanical complications from his MI including papillary muscle rupture, significant mitral regurgitation, ventricular septal defect, or obvious right ventricular infarction and/or intracardiac shunting, a transesophageal echocardiogram (TEE) was urgently performed to better evaluate for acute complications of MI. The TEE, instead revealed a long, mobile, serpiginous echodensity (0.7 cm in width) within the right and left main pulmonary artery with tertiary motion beyond the bifurcation consistent with acute saddle PE, and a dilated right ventricle with hypokinesis (Figure 2).

**Figure 3.**
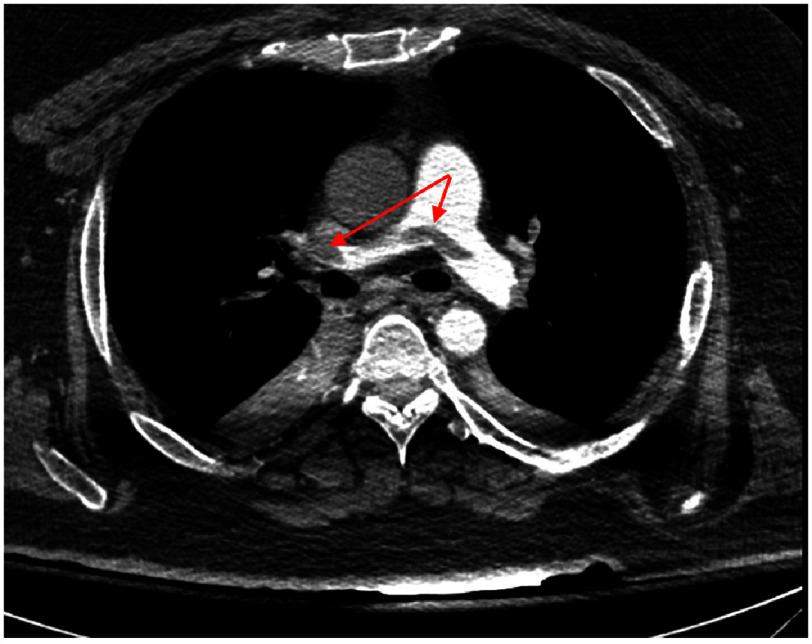
CTA of the chest revealing saddle thromboembolism in the main pulmonary artery and right upper lobar pulmonary artery (arrows).

Initial discussion ensued with the newly formed, multidisciplinary Pulmonary Embolism Response Team (PERT) at the time, with initial thoughts to administer tissue plasminogen activator (tPA), however, given severe hypoxia compounded by increasing pressor requirement obstructive shock, the decision was made to place the patient on VA-ECMO via right femoral access for management of hypoxia and hemodynamic support. After stabilization, the patient underwent CT angiography (CTA) of his chest which corroborated the saddle pulmonary embolus along with numerous filling defects in bilateral upper and lower lobar and segmental arteries (Figure 3).

Ultrasound of the lower extremities revealed a nonocclusive chronic thrombus in the right popliteal vein, and no evidence of deep venous thrombus in the left lower extremity veins. Repeat D-dimer levels increased to 7000’s ng/mL (normal range 0–243 ng/mL) over the next week, as well. The patient was maintained on aspirin, prasugrel was switched to cangrelor infusion for his original presenting inferior/posterior STEMI s/p mid RCA drug-eluting stent, in anticipation of any possible open heart procedure. For anticoagulation therapy, he was maintained on a heparin drip for his acute pulmonary embolism and the VA-ECMO circuit, without need for urgent interventional or surgical embolectomy. His condition gradually improved.

**Figure 4.**
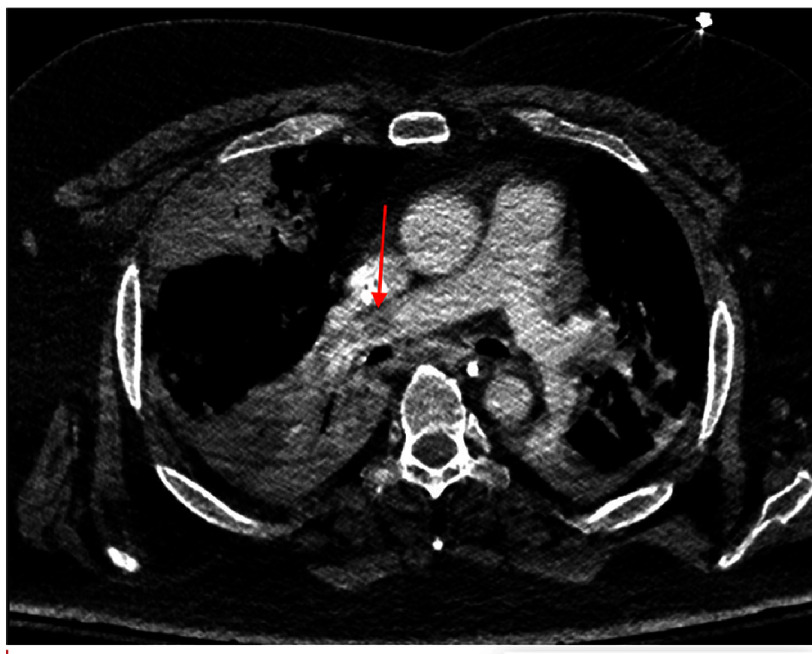
CTA of the chest showing that the previously seen saddle thrombus is not visualized, suggesting distal migration (arrow).

A repeat CTA of the chest 3 days later revealed distal migration of the saddle embolus, along with possible right upper lung lobe infarction (Figure 4).

**Figure 5.**
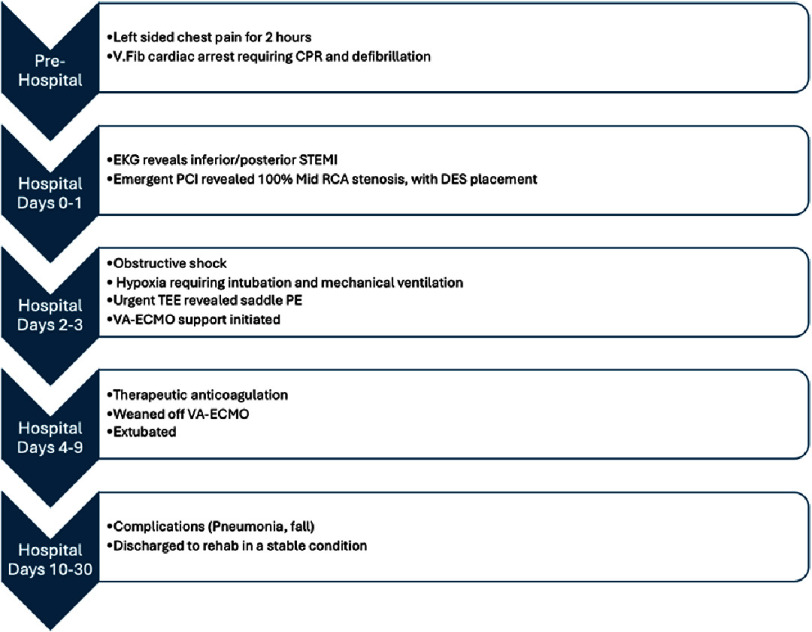
Visual summary of the case.

He was decannulated from VA-ECMO 9 days after its placement. His hospital course was further complicated by sepsis secondary to pneumonia and an in-hospital fall without traumatic injuries. He was eventually discharged to rehab in a stable condition a month after his admission, on clopidogrel and apixaban.

## Discussion

This case demonstrates the rare simultaneous occurrence of inferior/posterior STEMI followed shortly thereafter by acute PE and describes the latter’s diagnostic challenges. Their simultaneous occurrence poses diagnostic dilemmas as their symptoms and workup can overlap, often requiring further diagnostic testing for differentiation. The most common signs and symptoms found in acute PE are dyspnea, pleuritic chest pain, and tachypnea^2^. Symptoms such as lightheadedness or syncope could indicate the presence of RV dysfunction^3^. Common ECG findings in PE can include sinus tachycardia, right precordial T wave inversions, right bundle branch block, right axis deviation, and S waves in leads l and aVL^4^. In our case, the patient initially had a typical inferior/posterior STEMI promptly treated and stabilized by mid RCA drug-eluting stenting.

The co-occurrence of these two entities may result from shared risk factors or possibly a direct causal relationship. In a registry-based study, MI was found to be statistically associated with a 51% increased risk of venous thromboembolism and a 72% increased risk of PE^5^. In terms of pathophysiology, local changes in the cardiopulmonary circulation after MI’s can increase the risk of thrombus formation. This may occur due to stasis in the pulmonary circulation from heart failure related to left ventricular dysfunction, injury to the vascular endothelium, or activation of the coagulation system^5^. In fact, another registry noted that around 60% of the association between MI and venous thromboembolism/PE was mediated through immobilization or infection^6^. Our patient was found to have a chronic nonocclusive thrombus in the right popliteal vein, which was likely the source of embolization, given the acute inflammatory and hypercoagulable state post-MI. Additionally, he experienced immobilization, developed pneumonia (infection), and underwent CPR, all of which could have contributed to PE development.

Upon deterioration with hypoxia then shock a few days later, the leading diagnosis of some type of mechanical complication of acute MI was sought after. The presence of cardiogenic shock acutely post-MI usually signifies an underlying complication such as acute heart failure, acute papillary muscle ischemia or rupture and acute severe mitral regurgitation, ventricular free wall rupture, or ventricular septal defect, or acute right ventricular infarction^7^. Only during the urgent transesophageal echocardiogram performed in our patient to evaluate for an acute complication of MI, was an acute saddle pulmonary embolus incidentally discovered instead.

Acute PE can be missed in the inpatient setting. In a systematic review by Kwok et al., it was noted that up to a third of autopsies of patients who died in the intensive care unit had a PE that was not diagnosed previously. Additionally, about 12.4% of PE cases were initially misdiagnosed as ACS, adding to the overlap in their clinical presentation^1^. In our case, transesophageal echocardiogram would have missed unilateral or bilateral massive acute PE if it were not a saddle embolus.

In terms of antithrombotic management, this case illustrates the challenging balance between treating massive PE and minimizing bleeding risk in a patient on dual antiplatelet therapy (DAPT) after recent PCI. In the initial period, the patient required DAPT to reduce the risk of acute stent thrombosis after PCI, while massive PE with hemodynamic compromise necessitated full therapeutic anticoagulation. He was initially on aspirin and prasugrel for DAPT, and prasugrel was switched to cangrelor infusion should the patient undergo any open heart procedure such as surgical embolectomy. Heparin drip was also initially utilized for the same reason. Eventually, once the patient’s condition stabilized, prasugrel was switched to clopidogrel, a P2Y12 inhibitor that is associated with decreased bleeding risk as compared to prasugrel, making it more appropriate for combination therapy with an anticoagulant^8,9^.

VA-ECMO treatment has been proven to be beneficial as a treatment option in massive acute PE after an inferior/posterior STEMI, particularly for providing cardiopulmonary support until thrombolysis or spontaneous reperfusion are achieved^10^. In a review article by Kjaergaard et al., it was recommended to insert femoral sheaths prophylactically in massive PE patients before thrombolysis should a patient suffer cardiac arrest or cardiopulmonary collapse in the interim^11^.

In our case, VA-ECMO was selected over systemic thrombolysis due to multiple relative contraindications to fibrinolysis and the need for immediate hemodynamic support in a patient with refractory shock and hypoxia, while also preserving the option for later reperfusion therapy. A major concern was that if he had been administered thrombolysis and failed, that VA-ECMO and/or surgical embolectomy would not be offered given immediate, prohibitive post-thrombolysis bleeding risk. The recent PCI, recent traumatic/prolonged CPR, and being on dual antiplatelet therapy, also represented increased risk factors for thrombolysis. VA-ECMO here provided immediate hemodynamic and respiratory support while the patient was experiencing progressive hypoxia and refractory shock. It also preserved thrombolysis as a future option if needed. Additionally, VA-ECMO along with heparin therapy has been proven effective in massive PE. Our patient subsequently achieved reduced clot burden on VA-ECMO and heparin drip, ultimately without the need for thrombolysis or urgent interventional or surgical embolectomy^12^.

## What have we learned?

• In patients with acute MI or PE, clinicians should maintain a high suspicion for the coexistence of the other condition, especially when hypoxia or shock is unexplained.
• TEE can identify saddle pulmonary embolus in a patient too unstable to be transported for emergent CT angiography, but may miss unilateral or bilateral massive acute PE**.**
• Prompt diagnosis and a multidisciplinary approach are essential. VA-ECMO can be life-saving in select cases.
• Coexistent MI and PE are rare; more research is needed to determine optimal management strategies, including thrombolysis, urgent interventional or surgical embolectomy, and ECMO use.

## Ethics statement

In accordance with our institutional policy, case reports involving one or two patients do not require Institutional Review Board (IRB) approval. Therefore, this case report was exempt from IRB review. Per institutional requirements, the appropriate form was completed and submitted to the Privacy Office before preparation of this manuscript.

## Author statement

Conceptualization: Enad Haddad, Irfan Ahsan

Writing – Original Draft Preparation: Enad Haddad

Writing – Review & Editing: Gregary Marhefka, Enad Haddad, Irfan Ahsan, Sudeep Nugooru
